# The role of soft skills in academic achievement among physical education students: a mediation analysis

**DOI:** 10.3389/fpsyg.2026.1734218

**Published:** 2026-03-02

**Authors:** Quanhong Lu, Deke Liu, Yunmei Chai, Haipeng Yue, Song Yuan, Jingmiao Wang, Jianing Tian

**Affiliations:** Chengdu Sport University, Chengdu, China

**Keywords:** academic performance, cognitive abilities, mediation effect, physical education, soft skills

## Abstract

This study explores the role of soft skills in academic performance among physical education students, with a focus on the mediating effects of self-management, adaptability, and social interaction. A dual-path mediation model was proposed, wherein cognitive and innovative abilities are associated with academic achievement through two mediators: self-management and adaptability, and social interaction and teamwork. A survey of 627 undergraduate physical education students was conducted, and data were analyzed using structural equation modeling (SEM) and regression analysis. The results show that cognitive and innovative abilities are significantly related to academic performance both directly and indirectly. Specifically, self-management and adaptability, as well as social interaction and teamwork, were found to partially mediate these relationships. The findings underscore the critical role of soft skills in enhancing academic success, highlighting the indirect effect of cognitive abilities on academic performance through the development of soft skills. This study contributes to the existing literature by demonstrating the importance of soft skills in education and suggesting that educational institutions should integrate soft skills training into curricula to improve student outcomes. The implications for educational practice are discussed, emphasizing the need for a holistic approach to student development that combines cognitive and non-cognitive skills to foster both academic and professional success.

## 1 Introduction

In the knowledge economy era, the core mission of higher education has shifted from traditional knowledge transmission to the cultivation of students' overall competencies. Among these competencies, soft skills–such as thinking patterns, self-management, and interpersonal communication–are widely considered key factors that determine individual academic success and career development (Heckman and Kautz, 2012; Schneider and Preckel, 2017). In particular, the importance of soft skills is particularly prominent in physical education, a field that places high demands on practical ability and interpersonal interaction. Graduates of physical education programs are expected to play multiple roles, including that of a teacher, coach, and manager, all of which require excellent communication, collaboration, innovation, and self-regulation skills (Kondur et al., 2022). Therefore, exploring the structure and mechanisms of soft skills in this field is of pressing practical significance for optimizing talent development models and responding to national strategies such as “integration of sports and education” (Casali and Meneghetti, 2023; Insanistyo et al., 2025).

In this study, soft skills among physical education students are defined as three core dimensions: cognitive and innovative abilities, self-management and adaptability, and social interaction and teamwork skills. Previous research has confirmed a positive correlation between soft skills and academic performance (Keng, 2023; Loturco et al., 2022; Pan et al., 2025), yet these studies have several limitations: (1) in-depth studies specifically targeting physical education students are relatively scarce; (2) most studies focus on establishing the existence of a correlation, without exploring the underlying mechanisms of influence; (3) existing research often treats the various dimensions of soft skills as parallel constructs, overlooking the possibility of hierarchical structures or driving relationships within them. For example, can cognitive ability be considered a foundational “meta-skill” that facilitates the development of effective self-management strategies and successful social interactions? This question regarding the internal “structure” of soft skills remains an important theoretical gap and is central to the focus of this study (Gonçalves et al., 2024).

To uncover the internal mechanisms of soft skills, this study integrates two major theoretical frameworks: Self-Determination Theory (SDT) and Social Cognitive Theory (SCT). SDT posits that when the learning environment satisfies the basic psychological needs of autonomy, competence, and relatedness, intrinsic motivation is maximized, thus promoting positive learning behaviors (Ryan and Deci, 2000). SCT, on the other hand, emphasizes the dynamic interaction between personal factors (such as cognition), environmental factors (such as social support), and behavior, with self-efficacy playing a crucial role in linking cognition and behavior (Bandura, 1997).

Based on this, this study proposes a dual-path mediation model, with cognitive and innovative abilities serving as the core driver. It is hypothesized that cognitive and innovative abilities, as a foundational “meta-skill,” influence academic achievement through two parallel psychological and behavioral pathways. The first path is an intrinsic “self-regulation” pathway (from the SDT perspective): strong cognitive abilities allow students to plan and reflect on their learning more effectively, thereby fulfilling their deep psychological needs for autonomy and competence, which in turn enhances their self-management skills (Blegur, 2023). The second path is an external “social empowerment” pathway (from the SCT perspective): excellent cognitive abilities serve as the foundation for high-quality social interactions, enabling students to build a supportive environment through interaction with others, thus enhancing their academic self-efficacy, which is reflected in stronger social interaction skills.

Therefore, this study aims to go beyond a simple correlation analysis and provide an in-depth examination of the underlying mechanisms through which soft skills impact academic performance in physical education students. Specifically, this study addresses the following key research questions: How does cognitive and innovative ability influence academic achievement through self-management and adaptability, as well as social interaction and teamwork skills? To this end, we propose the following hypotheses:

Hypothesis 2: Cognitive and innovative ability is significantly associated with academic achievement.
Hypothesis 3: Self-management and adaptability mediate the relationship between cognitive and innovative ability and academic achievement.
Hypothesis 4: Social interaction and teamwork ability mediate the relationship between cognitive and innovative ability and academic achievement.

## 2 Methods

This study seeks to explore the relationship between soft skills and academic performance among physical education students, with a focus on identifying the mechanisms through which soft skills influence students' academic outcomes. The research design follows a quantitative approach, utilizing a structured questionnaire to collect data from a sample of physical education students. The study integrates multiple statistical methods to analyze the data, including descriptive statistics, correlation analysis, regression analysis, and structural equation modeling (SEM).

### 2.1 Participants and procedure

The participants were undergraduate students majoring in Physical Education at Chengdu Sport University. A total of 650 students were initially invited to participate. To ensure the accuracy of the data, a matching procedure was employed: students provided their student IDs during the survey, which were subsequently used to retrieve their official cumulative GPA from the university's Academic Affairs Office. After excluding 23 invalid responses, 627 valid questionnaires were retained (96.5% response rate). The final sample consisted of 351 male students (56.0%) and 276 female students (44.0%). The sample included 152 first-year (24.2%), 168 second-year (26.8%), 155 third-year (24.7%), and 152 fourth-year students (24.3%). All data were anonymized after the matching process to protect participant privacy.

### 2.2 Data collection

Data were collected through a combination of online and offline surveys. The questionnaire was designed to measure three core dimensions of soft skills: self-management and adaptability, cognitive and innovative abilities, and social interaction and teamwork skills. Each dimension was assessed using a Likert-type scale, with 38 items in total. The scale was developed based on a thorough literature review and expert consultations, ensuring its relevance and comprehensiveness in evaluating soft skills within the context of physical education students.

### 2.3 Measures

#### 2.3.1 Soft skills

The “Soft Skills Evaluation Scale for Physical Education Students in Higher Education” was used to assess soft skills. The scale underwent rigorous validation processes, including exploratory factor analysis (EFA) and confirmatory factor analysis (CFA), with a sample size of 627. The results indicated a robust theoretical structure and high internal consistency, with a Cronbach's alpha of 0.967 for the overall scale and subscales exceeding 0.91. See Tables 1, 2.

**Table 1:** Model fit coefficient (construct validity).

**Fitting index**	**Criterion for judgement**	**Actual value**	**Fit results**
**Absolute fitting index**			
CMIN/DF	< 3	1.063	Ample
RMR	< 0.05	0.027	Ample
GFI	>0.9	0.948	Ample
AGFI	>0.9	0.938	Ample
RMSEA	< 0.08	0.01	Ample
**Relative fitting index**			
NFI	>0.9	0.936	Ample
IFI	>0.9	0.996	Ample
TLI	>0.9	0.995	Ample
CFI	>0.9	0.996	Ample

**Table 2:** Factor loadings, AVE, and CR of each item.

**Way**			**Estimate**	**AVE**	**CR**
A11	< –	Autonomic learning	0.734	0.545	0.827
A12	< –	Autonomic learning	0.752		
A13	< –	Autonomic learning	0.732		
A14	< –	Autonomic learning	0.734		
A21	< –	Emotional regulation	0.697	0.523	0.766
A22	< –	Emotional regulation	0.707		
A23	< –	Emotional regulation	0.764		
A31	< –	Stress management	0.716	0.545	0.827
A32	< –	Stress management	0.741		
A33	< –	Stress management	0.767		
A34	< –	Stress management	0.727		
A41	< –	Acclimatization	0.718	0.542	0.826
A42	< –	Acclimatization	0.74		
A43	< –	Acclimatization	0.734		
A44	< –	Acclimatization	0.752		
B11	< –	Critical thinking	0.714	0.538	0.823
B12	< –	Critical thinking	0.75		
B13	< –	Critical thinking	0.736		
B14	< –	Critical thinking	0.734		
B21	< –	Problem-solving skills	0.716	0.535	0.821
B22	< –	Problem-solving skills	0.742		
B23	< –	Problem-solving skills	0.749		
B24	< –	Problem-solving skills	0.718		
B31	< –	Innovation ability	0.737	0.571	0.841
B32	< –	Innovation ability	0.721		
B33	< –	Innovation ability	0.744		
B34	< –	Innovation ability	0.816		
C11	< –	Communication skills	0.734	0.556	0.834
C12	< –	Communication skills	0.739		
C13	< –	Communication skills	0.761		
C14	< –	Communication skills	0.749		
C21	< –	Teamwork skills	0.774	0.604	0.82
C22	< –	Teamwork skills	0.773		
C23	< –	Teamwork skills	0.784		
C31	< –	Leadership	0.733	0.568	0.84
C32	< –	Leadership	0.743		
C33	< –	Leadership	0.76		
C34	< –	Leadership	0.776		

#### 2.3.2 Academic performance

Academic performance was operationalized as the students' cumulative GPA. As detailed in Section 2.1, this variable was obtained through official university records rather than self-report measures, ensuring a highly objective and reliable assessment of academic achievement.

#### 2.3.3 Control variables

Given that academic year and gender may influence students' soft skills and academic performance, these two demographic variables were included as control variables in the subsequent regression and mediation effect models.

### 2.4 Analytical methods

The data were analyzed using SPSS 26.0 and AMOS 26.0 software. The analysis process included the following steps:

(1) Descriptive Statistics and Correlation Analysis: Descriptive statistics were performed to examine the basic characteristics of the variables. Pearson correlation analysis was employed to explore the relationships between soft skills and academic performance.
(2) Regression Analysis: Hierarchical regression analysis was conducted to examine the direct effects of the independent variables (soft skills dimensions) and mediating variables (self-management, adaptability, social interaction, and teamwork skills) on academic performance. The regression model was structured in three steps: Step 1 included the control variables (gender, academic year), Step 2 incorporated the independent variables (soft skills dimensions), and Step 3 examined the impact of these variables on academic performance.
(3) Mediation Effect Testing: Structural equation modeling (SEM) was used to test the hypothesized mediation models. The Bootstrap method (5,000 samples) was applied to assess the significance of the mediation effects, with a 95% confidence interval (CI) for the indirect effects. A nonzero CI indicated a significant mediation effect.
(4) Model Fit: The model fit was evaluated using indices such as CMIN/DF, CFI, and RMSEA. Criteria for an acceptable model fit were as follows: CMIN/DF < 2, CFI > 0.90, and RMSEA < 0.08.
(5) Common Method Bias Test: Given that the soft skills data were collected from a single source, Harman's single-factor test was performed to assess the potential for common method bias.

### 2.5 Ethical considerations

This study was conducted in accordance with the ethical standards of the institutional research committee and the 1964 Helsinki Declaration and its later amendments. The research design followed the institutional guidelines of Chengdu Sport University for educational surveys involving students.

To ensure the protection of participants' rights, the following measures were implemented:

(1) Transparency and Consent: All participants were informed about the study's purpose and were assured that their responses would remain confidential. Informed consent was obtained from all individual participants included in the study.
(2) Voluntariness: Participation was strictly voluntary, and no incentives or coercion were used.
(3) Anonymization and Security: As the study required matching survey responses with official GPA records, student IDs were collected solely for this purpose. Once the data integration was complete, all identifying information was removed. The resulting dataset was fully anonymized and stored securely to protect participant privacy.

## 3 Results

### 3.1 Descriptive statistics and correlation analysis of variables

#### 3.1.1 Common method bias test

Since all soft skills data were collected via a single questionnaire, Harman's single-factor test was performed. The results indicated that the first factor explained 28.5% of the total variance. As this value is below the 40% criterion, it suggests that the relationships between cognitive abilities, self-management, and social interaction were not substantially distorted by common method variance.

#### 3.1.2 Descriptive statistics

Descriptive statistics were performed to summarize the characteristics of the study variables. The mean and standard deviation of each soft skills dimension and academic performance were calculated to assess the overall level and variability of the data. Table 3 presents the descriptive statistics for the soft skills dimensions and academic performance, including the mean values, standard deviations, and range of scores.

**Table 3:** Descriptive statistics for soft skills dimensions and academic performance.

**Variable**	**Mean**	**Standard deviation**	**Minimum**	**Maximum**
Self-management and adaptability	3.85	0.72	1.55	5.00
Cognitive and innovative abilities	3.32	0.68	1.70	4.90
Social interaction and teamwork	4.10	0.60	2.00	5.00
Academic performance (GPA)	84.21	6.78	63	97

The results revealed that students exhibited relatively high levels of soft skills, with the highest mean scores observed in the Social Interaction and Teamwork dimension (M = 4.10, SD = 0.60). This aligns with the practical and collaborative nature of physical education, where teamwork and communication are crucial. Conversely, the Cognitive and Innovative Abilities dimension received the lowest mean score (M = 3.32, SD = 0.68), indicating that students may struggle with more abstract cognitive tasks such as critical thinking and problem-solving.

Academic performance, as measured by GPA, exhibited a relatively wide range (M = 84.21, SD = 6.78), reflecting the diverse levels of achievement among students.

#### 3.1.3 Correlation analysis

**Figure 1:**
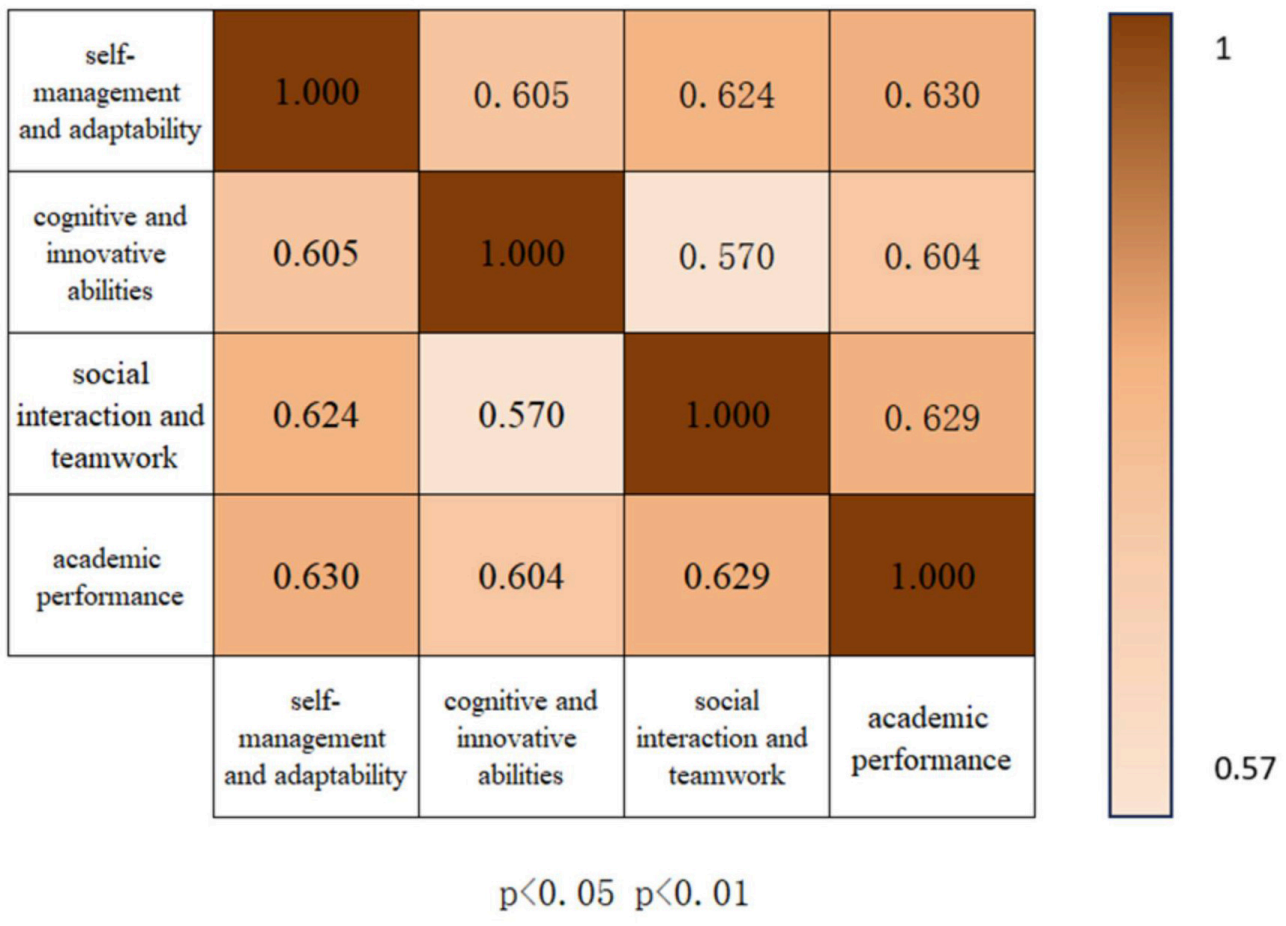
Pearson correlations between soft skills dimensions and academic performance.

Pearson correlation analysis was employed to examine the relationships between the soft skills dimensions and academic performance. The results showed strong positive correlations between all soft skills dimensions and academic performance (r values ranging from 0.604 to 0.630, all p < 0.01), indicating that higher levels of soft skills are associated with better academic performance. See Figure 1.

Specifically, Social Interaction and Teamwork (*r* = 0.630, *p* < 0.01) exhibited the strongest correlation with academic performance, suggesting that the ability to collaborate and communicate effectively plays a significant role in academic success. Cognitive and Innovative Abilities showed a moderate positive correlation with academic performance (*r* = 0.604, p < 0.01), suggesting that students who demonstrate stronger cognitive abilities also tend to perform better academically.

Additionally, Self-management and Adaptability (*r* = 0.618, *p* < 0.01) were positively correlated with academic performance, further supporting the importance of self-regulation in achieving academic success. These findings are consistent with the hypothesis that soft skills, including both cognitive and interpersonal abilities, are crucial predictors of academic achievement.

Further investigation into the relationships between the soft skills dimensions revealed significant positive correlations among them. Cognitive and Innovative Abilities were strongly correlated with Self-management and Adaptability (*r* = 0.605, *p* < 0.01) and Social Interaction and Teamwork (*r* = 0.570, *p* < 0.01). These results suggest that cognitive abilities not only directly affect academic performance but also contribute to the development of other soft skills, which in turn may enhance academic outcomes.

### 3.2 Regression analysis

**Figure 2:**
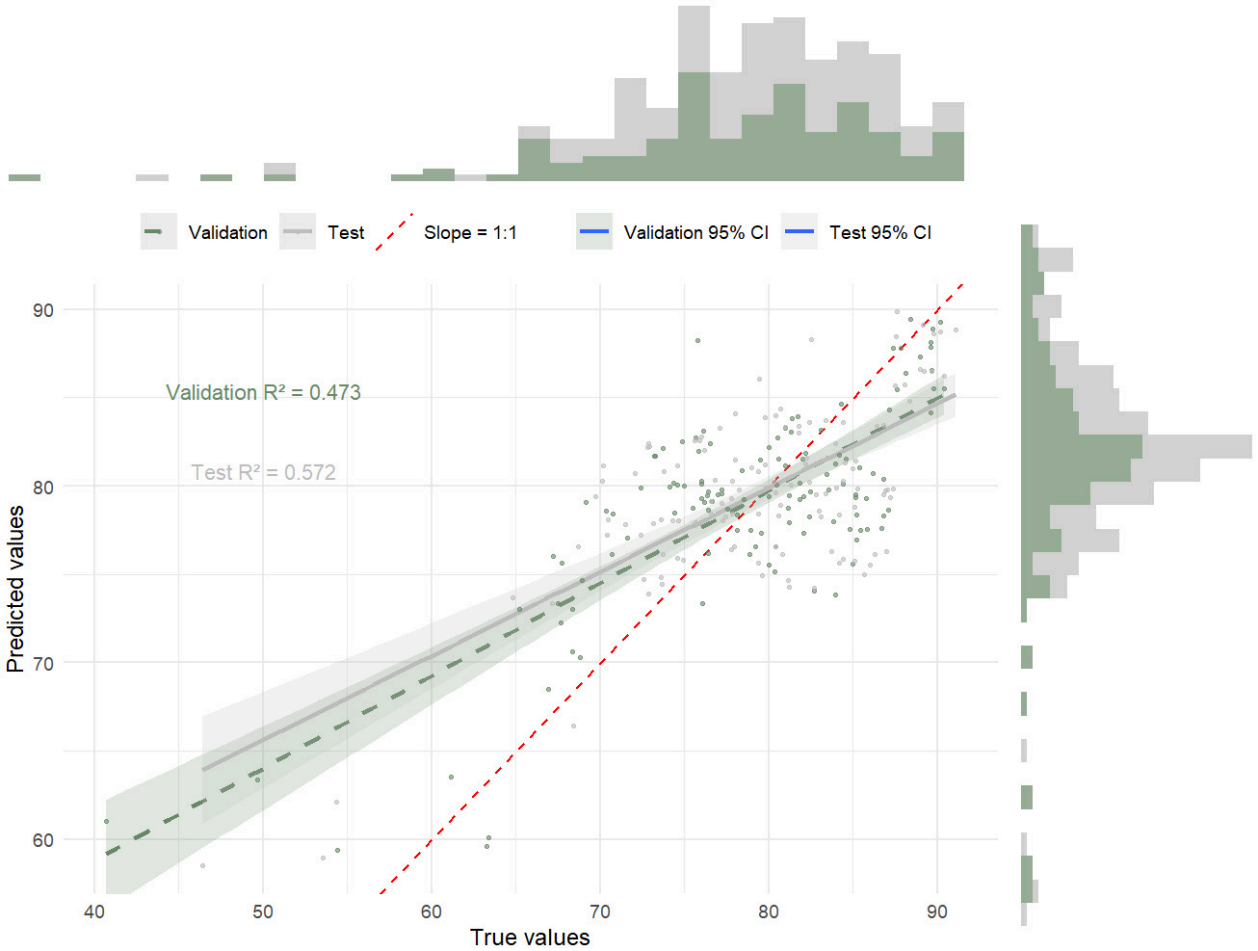
Hierarchical regression analysis of soft skills and academic performance.

To examine the direct effects of the soft skills dimensions on academic performance and explore the mediating roles of self-management, adaptability, social interaction, and teamwork, hierarchical regression analyses were performed. The analysis was conducted in three steps, progressively adding the independent and mediating variables to the regression model. See Figure 2.

#### 3.2.1 Control variables

In the first step, only the control variables (gender and academic year) were included in the model. The results showed that gender (β = 0.112, *p* > 0.05) and academic year (β = 0.092, p > 0.05) did not significantly predict academic performance, indicating that these demographic factors did not have a substantial effect on academic achievement in this study. Thus, the inclusion of these variables did not substantially alter the subsequent results.

#### 3.2.2 Direct effects of soft skills

In the second step, the three soft skills dimensions–Cognitive and Innovative Abilities, Self-management and Adaptability, and Social Interaction and Teamwork–were added to the model. The results showed that Self-management and Adaptability (β = 0.392, *p* < 0.01) and Social Interaction and Teamwork (β = 0.404, *p* < 0.01) significantly predicted academic performance. These findings highlight the importance of both intrapersonal (self-regulation) and interpersonal (teamwork) skills in enhancing students' academic outcomes.

Cognitive and Innovative Abilities (β = 0.247, *p* < 0.01) also significantly predicted academic performance, though to a lesser extent. This suggests that while cognitive abilities directly impact academic performance, their effect may be partially mediated through other soft skills, such as self-management and social interaction.

#### 3.2.3 Interaction effects and mediating variables

In the final step, the mediating roles of Self-management and Adaptability, and Social Interaction and Teamwork were tested by including the interaction terms between cognitive abilities and the mediating variables. The results revealed that both Self-management and Adaptability (β = 0.477, *p* < 0.01) and Social Interaction and Teamwork (β = 0.467, *p* < 0.01) significantly predicted academic performance when cognitive and innovative abilities were included as predictors.

This suggests that cognitive abilities indirectly influence academic performance through their effects on students' self-regulation and teamwork skills. In fact, Self-management and Adaptability and Social Interaction and Teamwork served as significant mediators of the relationship between Cognitive and Innovative Abilities and academic performance.

#### 3.2.4 Interpretation of results

The results of the regression analyses confirm that soft skills are significant predictors of academic performance among physical education students. Self-management and Adaptability and Social Interaction and Teamwork have the strongest direct effects on academic performance, underlining the importance of both intrapersonal and interpersonal skills for academic success. Cognitive and Innovative Abilities also directly influence academic performance, but their impact is somewhat mediated by the other soft skills dimensions.

Furthermore, the regression analysis in Step 3 demonstrated that the effect of cognitive and innovative abilities on academic performance is partially mediated by Self-management and Adaptability and Social Interaction and Teamwork. These findings suggest that enhancing students' cognitive abilities alone may not be sufficient for academic success; developing complementary soft skills, such as self-regulation and teamwork, is crucial.

### 3.3 Mediation effect model testing

**Figure 3:**
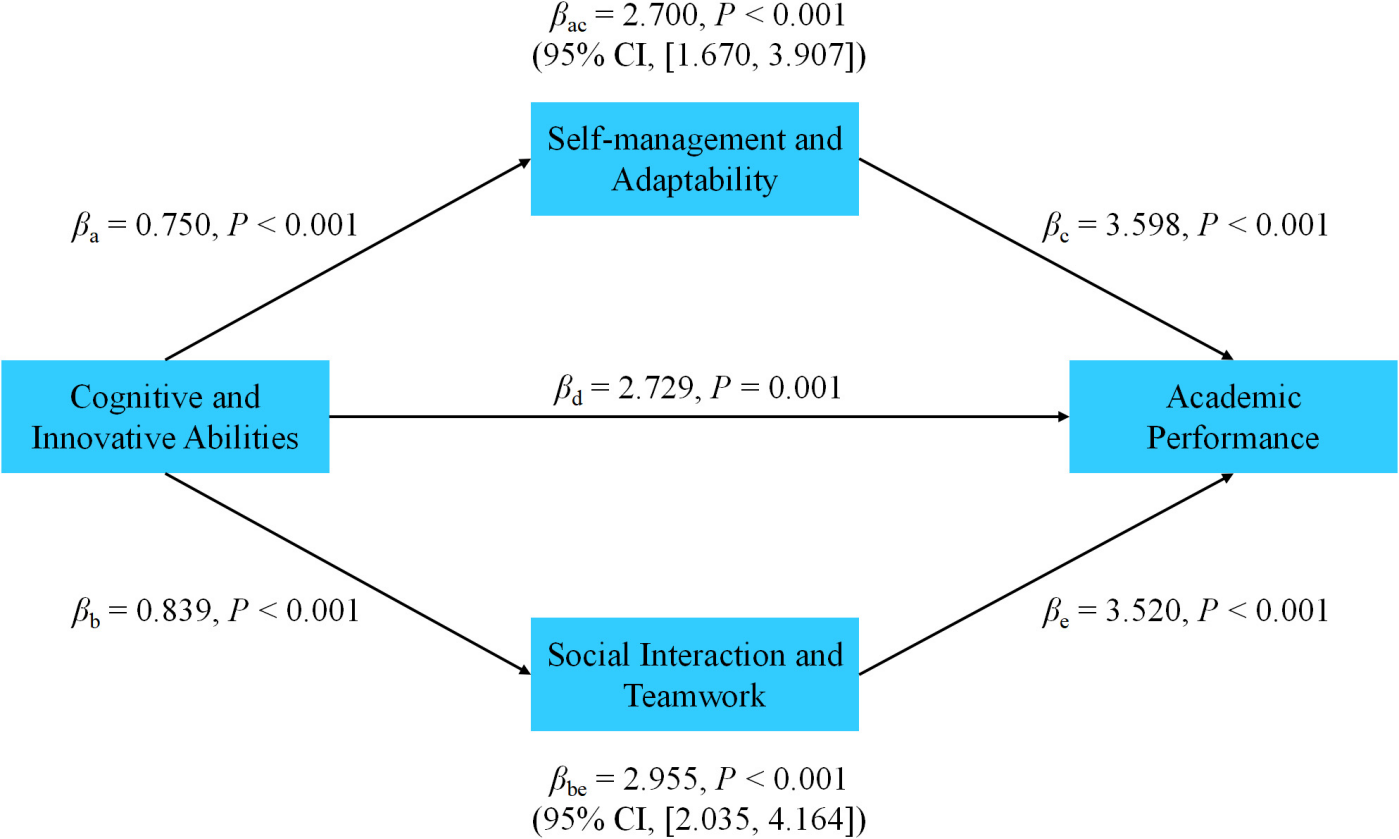
Standardized path coefficients of the dual-mechanism model.

This section investigates the mediation effects of soft skills in the relationship between cognitive and innovative abilities and academic performance. The Bootstrap method was used to assess the significance of the indirect effects, particularly focusing on the mediating roles of self-management and adaptability, and social interaction and teamwork, To provide a clearer interpretation, both unstandardized and standardized indirect effects β are reported in Table 4. See Figure 3.

**Table 4:** Mediation effect results (*N* = 627).

**Way**	**Effect**	**Effect**	**SE**	**LLCI**	**ULCI**
Cognitive and innovative abilities-Self-management and adaptability- school achievement	Indigo effect	0.198	0.032	0.133	0.258
	Direct effect	0.392	0.059	0.502	0.734
	Gross effect	0.59	0.059	0.814	1.047
Cognitive and innovative abilities-social interaction and teamwork ability-academic achievement	Indigo effect	0.186	0.029	0.127	0.242
	Direct effect	0.404	0.058	0.524	0.751
	Gross effect	0.59	0.059	0.814	1.047

#### 3.3.1 Model fit indices

The model fit was evaluated using standard fit indices, and the results indicated that the model fit the data well. The key fit indices are as follows:

- CMIN/DF = 1.89, which is below the threshold of 2, indicating a good model fit.
- CFI = 0.956, which exceeds the acceptable threshold of 0.90, indicating a good fit.
- RMSEA = 0.045, which is well below the cutoff value of 0.08, suggesting an acceptable fit.

These indices indicate that the proposed mediation model provides a good representation of the data and that the relationships between the variables are appropriately modeled.

#### 3.3.2 Interpretation of mediation effects

The results from the mediation analysis demonstrate that cognitive and innovative abilities significantly influence academic performance, both directly and indirectly through the mediation of soft skills. The indirect effects were analyzed using the Bootstrap method, and the results are as follows:

- Cognitive and Innovative Abilities → Self-management and Adaptability → Academic Performance

The indirect effect of cognitive and innovative abilities on academic performance through self-management and adaptability was 2.700 (95% CI: [1.670, 3.907]), which is statistically significant. This suggests that students with stronger cognitive abilities are more likely to manage their learning processes effectively and adapt to challenges, which in turn enhances academic performance. This finding is in line with Self-Determination Theory (SDT), which posits that the fulfillment of psychological needs such as autonomy and competence promotes intrinsic motivation and better learning outcomes.

- Cognitive and Innovative Abilities → Social Interaction and Teamwork → Academic Performance

The indirect effect of cognitive and innovative abilities on academic performance through social interaction and teamwork was 2.955 (95% CI: [2.035, 4.164]), which was also significant. This indicates that cognitive abilities promote better social interaction and teamwork skills, which contribute to enhanced academic performance. Strong cognitive abilities, such as critical thinking and problem-solving, help students interact more effectively with peers and instructors, leading to improved collaboration and support, which ultimately boosts academic performance. This finding supports Social Cognitive Theory (SCT), which highlights the importance of social interactions and self-efficacy in learning.

#### 3.3.3 Total indirect effect

The total indirect effect, combining the two mediation pathways (self-management and adaptability, and social interaction and teamwork), was 5.655 (95% CI: [4.199, 7.276]), indicating that soft skills significantly mediate the relationship between cognitive and innovative abilities and academic performance. This result underscores the importance of developing soft skills alongside cognitive abilities to maximize academic success.

#### 3.3.4 Implications of the mediation effects

The results from the mediation analysis demonstrate that cognitive and innovative abilities are significantly associated with academic performance, both directly and indirectly through the statistical mediation of soft skills. Specifically, stronger cognitive abilities are linked to enhanced social interaction and teamwork, which in turn are positively related to academic achievement.

## 4 Discussion

The findings of this study provide valuable insights into the role of soft skills in academic performance among physical education students. The results support the hypothesis that Cognitive and Innovative Abilities significantly contribute to academic performance, both directly and indirectly, through their mediation by Self-management and Adaptability and Social Interaction and Teamwork. These findings have important theoretical and practical implications, which are discussed below.

### 4.1 Core driving role of cognitive and innovative abilities

One of the most important findings of this study is the confirmation of the core central role of “cognitive and innovative abilities” within the soft skills system. In the conceptual model, it is positioned as a foundational variable linked to other dimensions. This result is highly consistent with the central idea in Social Cognitive Theory (SCT), which posits that “cognition precedes behavior”. For physical education students, critical thinking, information literacy, and problem-solving skills are foundational to digesting complex sports science theories, designing scientific training programs, and reflecting effectively in teaching practice. Unlike simple knowledge memorization, these higher-order cognitive abilities equip students with the ability to “learn how to learn,” enabling them to proactively and efficiently tackle various academic challenges, thereby directly enhancing academic performance.

### 4.2 Theoretical deconstruction of the mediation mechanisms: internal transformation and external empowerment

The dual-path mediation model identified in this study elucidates how cognitive and innovative abilities are translated into academic success through two distinct yet complementary psychological and behavioral mechanisms. This confirms that cognitive ability functions as a foundational “meta-skill” that drives academic performance by optimizing both internal regulatory processes and external social interactions.

#### 4.2.1 The intrinsic “self-regulation” pathway: an SDT perspective

The first pathway (Cognitive Abilities — Self-management — Academic Achievement) represents an internal transformation from cognitive potential to autonomous action. According to Self-Determination Theory (SDT), the quality of a student's engagement depends on the satisfaction of basic psychological needs.

- Cognitive Mastery as a Prerequisite for Autonomy: Higher-order cognitive abilities allow students to engage in Organismic Integration, a process where external academic requirements are internalized into personal values. Students with robust cognitive capacities can deconstruct complex academic tasks, enabling them to exercise autonomous regulation over their learning schedules and training regimens.
- Competence Satisfaction and Strategic Persistence: When students apply innovative thinking to navigate challenges in sports practice, they experience a profound sense of competence satisfaction. This psychological fulfillment acts as a catalyst for sustained self-management and adaptability, shifting the learning process from passive compliance to active monitoring and strategic adjustment, which ultimately yields superior academic outcomes.

#### 4.2.2 The extrinsic “social empowerment” pathway: an SCT perspective

The second pathway (Cognitive Abilities — Social Interaction — Academic Achievement) illustrates how personal cognitive factors optimize the learning environment to foster performance. This is deeply rooted in the Triadic Reciprocal Determinism of Social Cognitive Theory (SCT).

- Cognitive Foundations of Interpersonal Agency: In the highly collaborative field of physical education, cognitive skills—such as perspective-taking and logical expression—serve as the bedrock of high-quality social interactions. Students with stronger cognitive abilities are more effective in navigating the social complexities of team-based sports and peer-led coaching, allowing them to secure critical social resources and emotional support.
- Collective Intelligence and Academic Self-Efficacy: These positive interaction experiences are not merely informational but serve as mastery experiences and social persuasion. According to SCT, such experiences significantly enhance a student's academic self-efficacy—the robust belief in one's capability to complete tasks through collaborative efforts. This pathway reveals the transition from “individual intelligence” to “collective intelligence,” whereby the student strategically utilizes social empowerment to maximize their academic potential.

### 4.3 Practical implications

The findings of this study offer several actionable strategies for educators and policymakers to optimize talent development models in physical education (PE) within higher education:

#### 4.3.1 Strategic shift to competency-based curricula

Educational institutions should transition from a traditional “knowledge-based” approach to a “competency-based” model, positioning cognitive and innovative abilities as the foundational core.

(1) Actionable Strategy (Project-Based Learning): Educators should systematically integrate Project-Based Learning (PBL) and case-study methods into PE curricula. For instance, rather than focusing solely on athletic techniques, students could be tasked with designing and managing real-world community sports projects or organizing campus-wide athletic events. Such high-challenge projects require the simultaneous application of critical thinking, innovative problem-solving, and social coordination.
(2) Instructional Focus: Teaching should move beyond the “what” to emphasize the “why” and “how,” making the cultivation of complex problem-solving skills a core objective across all courses.

#### 4.3.2 Implementation of a “dual approach” to soft skills training

To effectively bridge the gap between cognitive understanding and behavioral application, a dual-pathway approach is recommended to foster both self-regulation and interpersonal agency.

- Actionable Strategy (Mentorship Programs): Institutions can implement a “mentor + growth partner” system. In this model, mentors guide students in utilizing their cognitive planning skills to develop personalized growth blueprints, while peer-to-peer partnerships provide a safe environment to practice adaptability and self-management.
- Platform Creation: Students must be provided with practical platforms to apply cognitive abilities to teamwork scenarios, such as leadership roles in sports teams or collaborative teaching internships, which allow for real-world practice of conflict resolution and communication.

#### 4.3.2 Contextual alignment with national and cultural strategies

Policymakers must align soft skills training with the specific institutional and cultural landscape of Chinese higher education.

- Policy Integration: The findings support China's national strategy for the “integration of sports and education” (体教融合), which demands a holistic approach to student development. Policymakers should encourage PE programs to evolve from being exclusively skill-oriented to models that value both cognitive depth and non-cognitive competencies.
- Cultural Leverage: Recognizing the collectivist orientation in Chinese sports culture—where team honor and interpersonal harmony are prioritized—educators can leverage this to emphasize “Social Interaction and Teamwork” as a driver of academic success. Curricula should be designed to help students navigate the balance between the rigorous self-discipline required for training and the complex social coordination required for professional coaching and teaching practices.

### 4.4 Limitations and future directions

Although this study has provided valuable insights, it still has some limitations. First, this study employed a cross-sectional design, meaning all data were collected at a single point in time. Consequently, while our mediation model is theoretically grounded in Self-Determination Theory and Social Cognitive Theory, it cannot confirm definitive causal relationships between soft skills and academic performance. The paths identified in our model should be interpreted as statistical associations rather than causal chains. Future studies should adopt a longitudinal tracking design to measure students' soft skills and academic achievement at different time points, which would allow for a more reliable examination of the causal directions suggested by this study. Second, the generalizability of the findings is limited by the sample characteristics. The participants were drawn from a single specialized physical education institution in China. Therefore, the conclusions might not be fully representative of PE students in other educational settings, such as sports departments within comprehensive universities, where academic environments and curriculum structures differ. Furthermore, because the study was conducted within a specific cultural and educational context, the results may not be true for PE students in other countries. Future research should include more diverse, multi-institutional, and international samples to validate the cross-cultural applicability of the model. Third, while we officially obtained GPA records to ensure data accuracy and conducted a common method bias (CMB) test, the soft skills data were still based on student self-reports. Although the Harman's single-factor test suggested that CMB was not a significant concern in this study, the self-report nature of the survey might still introduce subjective bias. Future studies could incorporate multi-source data, such as teacher or coach evaluations, to further enhance the objectivity of soft skill measurements. Lastly, this study focused on the internal mediation mechanisms but did not address potential moderating variables. Future research could further explore whether contextual factors, such as specific teaching styles or the difficulty level of courses, moderate the impact pathways of soft skills on academic outcomes.

## Data Availability

The original contributions presented in the study are included in the article/supplementary material, further inquiries can be directed to the corresponding authors.
